# Correlations between Electro-Diagnostic Findings, the Severity of Initial Infection, and the Rehabilitation Outcomes among COVID-19 Patients

**DOI:** 10.3390/biology11020277

**Published:** 2022-02-10

**Authors:** Sheer Shabat, Zeev Meiner, Jeanna Tsenter, Isabella Schwartz, Sigal Portnoy

**Affiliations:** 1 Faculty of Medicine, Hebrew University of Jerusalem, Jerusalem 91905, Israel; sheer.benyehuda@mail.huji.ac.il (S.S.); meiner@hadassah.org.il (Z.M.); tsenterj@hadassah.org.il (J.T.); IsabellaS@hadassah.org.il (I.S.); 2 Department of Physical Medicine and Rehabilitation, Hadassah Medical Center, Jerusalem 9765418, Israel; 3 Department of Occupational Therapy, Sackler Faculty of Medicine, Tel Aviv University, Tel Aviv 6997801, Israel

**Keywords:** electromyography, EMG/NCS, WHO clinical progression scale, functional independence measure, pain, rehabilitation

## Abstract

**Simple Summary:**

Patients with Coronavirus-2019 (COVID-19) often have reduced muscle strength and loss of sensory function. We examined several properties of the function of the nerve located at the arm and leg of 19 COVID-19 hospitalized patients before and after their rehabilitation period. We also evaluated the severity of their illness, their gait, muscle strength, and level of disability. We isolated several factors in the function of their nerves, which can be used to predict their prognosis and rehabilitation outcomes. Our findings are important since clinicians can use examinations of nerve function at early stages of the illness in order to devise an optimal treatment plan for the patient, thereby reducing the hospitalization period and promoting patient’s independence.

**Abstract:**

Patients with Coronavirus-2019 (COVID-19) manifest many neuromuscular complications. We evaluated the correlations between electromyography and nerve conduction measurements among COVID-19 patients and the severity of the initial infection, as well as the rehabilitation outcomes, and searched for the factors which best predict the rehabilitation outcomes. A total of 19 COVID-19 patients (16 men; mean ± SD age 59.1 ± 10.4), with WHO clinical progression scale of 6.8 ± 2.3, received rehabilitation for 3.9 ± 2.5 months. The Functional Independence Measure (FIM), the 10 m walk test, the 6 minute walk test, and grip force were collected before and after the rehabilitation period. Motor Nerve Conduction (MNC), Sensory Nerve Conduction (SNC) and electromyographic abnormalities were measured. All of the MNC measures of the median nerve correlated with the WHO clinical progression scale and duration of acute hospitalization. The MNC and SNC measures correlated with the rehabilitation duration and with FIM at discharge. The MNC distal latency of the median and the peroneal nerves and the MNC velocity of the median and tibial nerves predicted 91.6% of the variance of the motor FIM at discharge. We conclude that nerve conduction measurements, especially in COVID-19 patients with severe illness, are important in order to predict prognosis and rehabilitation outcomes.

## 1. Introduction

Neurological manifestation appear in approximately 36% of patients with coronavirus disease 2019 (COVID-19) [1,2]. Many of them manifest multiple neuromuscular complications, including widespread involvement of the peripheral nervous system, as well as the neuromuscular junction and muscles [3,4,5]. Some peripheral injuries are attributed to prolonged immobilization in intensive care units [6,7,8]. However, since both central and peripheral nervous system dysfunctions may be present, etiologies supported by inflammatory or autoimmune studies are also considered [6]. It was also suggested that peripheral neuropathies might result from neurotoxic side effects of drugs applied to treat COVID-19, e.g., Daptomycin, Linezolid, and Lopinavir [9].

Previous studies that quantified the electromyography and nerve conduction among COVID-19 patients reported that most of the injuries were either Critical Illness Polyneuropathy (CIP) or Critical Illness Myopathy (CIM) or both [10,11]. Cabañes-Martínez et al. diagnosed 11 patients electro-diagnostically with either CIP or CIM out of 225 COVID-19 patients, with the latter being present in the majority [12]. Hameed et al. reported the electro-diagnostic findings of 18 COVID-19 patients, in which the majority were consistent with a myopathy (82%) and 5 of them also had a concurrent axonal neuropathy [7]. Santiago-Pérez, et al. reported the electrophysiological results of 22 COVID-19 patients [13]. Myopathy was diagnosed in 17 patients (77.3%) and polyneuropathy in 4 (18.2%). Focal neuropathies were diagnosed in 12 patients (54.6%), with a total of 19 affected nerves. Common peroneal nerve lesions at the fibular head (68.4%) and ulnar nerve lesions at the elbow level (21.1%) were the most frequent locations. These studies provide a strong basis of evidence for electrophysiological abnormalities in COVID-19 patients that should be considered when a rehabilitation treatment plan is devised for inpatients and outpatients [14].

Several studies reported the effect of multidisciplinary rehabilitation programs for COVID-19 patients manifesting long term sequelae, including patients with neuromuscular involvement, such as CIM or CIP [15]. In France, COVID-19 patients (*n* = 100) received early inpatient rehabilitation [16]. The authors reported positive motor progression and negative correlation between grip strength and the number of days spent in intensive care, both at admission and discharge. In Philadelphia, rehabilitation outcomes of COVID-19 patients (*n* = 43) were compared to non–COVID-19 inpatient rehabilitation patients (*n* = 247) with impairment codes that were frequent for the COVID-19 patients [17]. While COVID-19 patients had greater deficits at admission, they eventually reached similar functional outcomes compared with the non–COVID-19 patients. In Brazil, COVID-19 patients (*n* = 27) who received multidisciplinary rehabilitation significantly improved their muscle strength, ambulation ability and functional independence [18]. Moreover, the duration of treatment positively correlated with the evolution of the Functional Independence Measure (FIM) score. In Turkey, grip strength at discharge was similar between COVID-19 patients (*n* = 18) who received range of motion exercises and neuromuscular electrical stimulation, and COVID-19 patients (*n* = 17) who received standard care [19].

While there are several studies that quantified the electromyography and nerve conduction among COVID-19 patients as well as studies that quantified their rehabilitation outcomes, there are no studies investigating the correlations between the electrophysiological findings of COVID-19 patients with neuromuscular involvement and the severity of the acute infection, as well as the rehabilitation outcomes. Our study objectives were (a) to evaluate the correlations between electromyography and nerve conduction measurements among COVID-19 patients and the severity of the initial infection, as well as the rehabilitation outcomes, and (b) to find the factors that best predict the rehabilitation outcomes.

## 2. Methods and Materials

**Figure 1:**
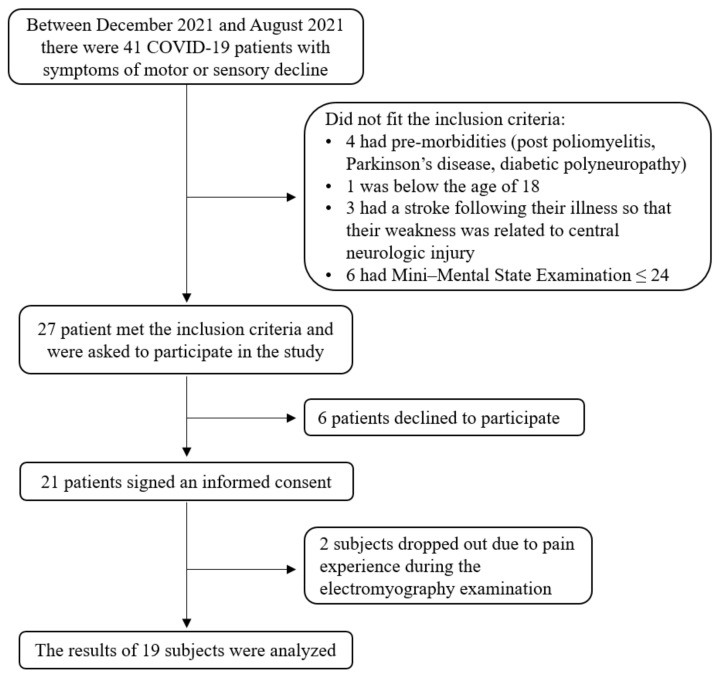
The recruitment of coronavirus disease 2019 (COVID-19) patients for this study.

Population: We recruited post-acute COVID-19 patients. Data were collected between December 2020 and August 2021. The recruitment process is described in Figure 1. All of the subjects who agreed to participate in the study and fitted the inclusion criteria were prospectively recruited. Inclusion criteria were: adults above 18 years of age, inpatients and outpatients referred to the rehabilitation department to a rehabilitation program that focused on respiratory and endurance physiotherapy, executive functions practice, psychological treatment, and support groups (referral occurs when at least 2 of the following are found: reduced endurance, motor impairment, functional impairment, cognitive impairment, or mental impairment), Mini–Mental State Examination (MMSE) score above 24, able to understand and sign an informed consent form. Exclusion criteria: pre-morbidity of peripheral neuropathy, musculoskeletal disease, or mental disorder, dementia. Ethical approval was granted by the Hadassah medical center Helsinki committee pretrial (approval number 0943-20-HMO). All of the subjects read and signed an informed consent form.

Tools and protocol: Subject demographics and rehabilitation measures were acquired from the hospital records. Data included were age, sex, comorbidity of diabetes, duration in acute hospitalization, duration of ventilation, steroids (yes/no), and duration of rehabilitation. Additionally, the World Health Organization (WHO) clinical progression scale for COVID-19 patients [20] was used. The scale ranges from ‘0′ (uninfected) to ‘10′ (dead) and the values of 1–9 are divided to group of ambulatory mild disease (1–3 that differ by factors of asymptomatic or not, independent or not), hospitalized moderate disease (4–5 that differ by with or without oxygen by mask or nasal prongs), and hospitalized severe disease (6–9 that differs by invasive or non-invasive mechanical ventilation, levels of pO_2_/FiO_2_ and SpO_2_/FiO_2,_ need of vasopressors, dialysis or ECMO) [20].

Each subject marked the perceived level of pain, which is one of the clinical features of COVID-19 [21]) on a Visual Analogue Scale (VAS) rated from ‘0′ (no pain) to ‘10′ (worst imaginable pain). Clinical evaluation distinguished between nociceptive (musculoskeletal) and neuropathic pain types [22]. The following measures were taken at admission and at discharge: the FIM, which is comprised of 18 items, grouped into motor and cognition subscales. Each item is scored on a scale of ‘1′ (total assistance or not testable) to ‘7′ (performs independently in a safe and timely manner). Higher score reflects high independence in activities of daily living. The Minimal Clinically Important Difference (MCID) of the FIM was determined for post stroke patients as 3 for cognitive FIM, 17 for motor FIM, and 22 for total FIM [23]. Another measure was the 10 m walk test, where the time (in seconds) that it takes the subject to complete a 10 m walk on paved floor is measured. The gait speed (in m/s) can be calculated by dividing 10 m with the walking duration. The MCID for both geriatric and post stroke population is 0.1 m/s [24]. We also performed the 6 minute walk test (6MWT) that test endurance as the subject is asked to walk on a paved path for 6 min and the path length is measured in meters. The MCID in the geriatric population is 50 m [24]. Additionally, grip force was measured using the hand held Jamar dynamometer for both hands. The measurement is normalized by normal data of similar healthy age groups [25].

Finally, a single electrophysiological assessment was performed following the patient’s admission to the rehabilitation department using a 2-channel electromyography (EMG) device (VikingQuest, 2018 model, Natus Medical Inc., Orlando, FL, USA). Mean time from acute COVID-19 diagnosis to electrophysiological assessment was 5.8 ± 2.2 months. Motor Nerve Conduction (MNC) parameters of distal latency (in ms), amplitude of the action potential (in mV), and conduction velocity (in m/s) were assessed at the wrists and ankles. Antidromic Sensory Nerve Conduction (SNC) parameters of action potential amplitude and conduction velocity of the sural and ulnar nerves were also acquired.

Statistical analysis: Statistical analyses were performed using SPSS 27.0 (SPSS Chicago, IL, USA). Data are provide in the Supplementary Material. Non-parametric tests were performed due to the small sample size. Descriptive statistics is therefore presented as median and interquartile range. The Wilcoxon signed-rank test was used to test for differences in outcome measures collected in admission and outcome measures collected at discharge. The effect size, *r*, was calculated using the following equation [26]:

$$r=\frac{z}{\sqrt{N}}$$

Additionally, the Mann-Whitney test was used to test for differences between subjects with and without myopathy. Correlations were performed using the Spearman’s rank test and stepwise linear regression was used to explain the variance of the duration of rehabilitation. *p* < 0.05 was considered statistically significant.

## 3. Results

### 3.1. Demographic Data (Descriptive Statistics)

A total of 19 subjects (16 men and 3 women; mean ± SD age of 59.1 ± 10.4) participated in this study. The mean ± SD of their WHO clinical progression scale was 6.8 ± 2.3 (range 2 to 9). Specifically, 3 subjects (15.8%) were diagnosed with ambulatory mild disease, 1 subject (5.3%) was hospitalized with moderate disease, and 15 subjects (78.9%) were hospitalized with severe disease, according to the WHO clinical progression scale. Moreover, 10 (52.6%) subjects had diabetes. A total of 16 (84.2%) subjects were admitted to acute hospitalization for a duration of 6.1 ± 2.8 weeks; 14 (73.7%) subjects were intubated and ventilated for a duration of 20.1 ± 8.3 days; 16 (84.2%) subjects received steroids. The duration of the rehabilitation was 3.9 ± 2.5 months. A total of 4 (21.0%) subjects reported musculoskeletal pain with VAS scores of 5.8 ± 2.9 and 8 (42.1%) subjects reported neuropathic pain with VAS scores of 6.6 ± 1.9. Five of the eight subjects who complained about neuropathic pain received neuropathic treatment.

### 3.2. Electrophysiological Findings

According to our EMG and electrophysiological examination, 15 subjects (78.9%) were diagnosed with pathologies, as follows: 4 subjects (21.1%) had Sensorimotor polyneuropathy compatible to critical illness polyneuropathy, 4 subjects (21.1%) had mononeuritis multiplex with 3 or more nerves involved, 4 subjects (21.1%) had radicular injuries (mostly lumbosacral level), 2 subjects (10.5%) had plexopathy, 1 with severe bilateral brachial plexopathy and 1 had an involvement of left upper lumbar and brachial plexus. Finally, 2 subjects (10.5%) showed focal neuropathies, 1 had right ischemic tibial and peroneal neuropathy as a result of deep vein thrombosis and the other had left lateral femoral cutaneous neuropathy and right superficial peroneal neuropathy. EMG results of 9 patients (47.4%) showed characteristics consistent with proximal critical illness myopathy, including spontaneous activity of positive sharp waves and fibrillations, as well as, short duration and small amplitude of Motor Unit Action Potentials (MUAP) with early recruitment. All 9 patients also had 1 of the aforementioned neuropathies. Frequencies of pathological finding in the electrophysiological measurements are presented in Table 1. Since we found no between-group differences in all of the outcome measures between subjects with myopathy (*n* = 9) and subjects without myopathy (*n* = 10), further correlation analyses were performed for the entire sample size.

### 3.3. Rehabilitation Outcomes

The outcome measures of FIM, grip force, 6MWT, and 10MWT, were improved following the rehabilitation (Table 2). Only 2 subjects (10.5%) improved their cognitive FIM scores by more than the MCID, and 4 (21.1%) improved their motor and total FIM scores by more than the MCID. A total of 16 subjects (84.2%) improved their 10MWT by more than the MCID; additionally, 15 (78.9%) subjects improved their 6MWT by more than the MCID.

### 3.4. Correlations between the WHO Clinical Progression Scale and Duration of Acute Hospitalization and Electrophysiological Factors

Correlations between the WHO clinical progression scores and duration of acute hospitalization (in weeks) and electrophysiological factors are presented in Table 3. The WHO clinical progression scores showed moderate to high correlation with MNC latency, velocity and amplitude of the median nerve and moderately correlated with the MNC amplitude of the tibial nerve. No correlations were found between the WHO clinical progression scores and the SNC measurements. In our study, only the median nerve latency and amplitude correlated with the duration of acute hospitalization.

### 3.5. Correlations between the Rehabilitation Durations Functional and Electrophysiological Measurements

Correlations between the functional and electrophysiological measurements are presented in Table 4. Our main findings show strong correlations between the MNC amplitude of all tested nerves and the duration of rehabilitation, as well as the motor FIM at discharged (but for the ulnar nerve). Greater amplitude was correlated with a shorter duration and higher FIM at discharge. Similar correlations were found between sensory conduction velocity of the median and ulnar nerves and the duration of rehabilitation and FIM at discharge.

### 3.6. Regression Analysis

A total of 4 parameters explained 91.6% of the variance of the motor FIM at discharge (*p* < 0.001): MNC latency of the median and the peroneal nerves and the MNC velocity of the median and tibial nerves. The fitted regression model was Motor FIM at discharge = 84.851 − 5.094 (MNC latency of the median nerve) − 4.023 (MNC latency of the peroneal nerve) + 1.137 (MNC velocity of the median nerve) − 0.391 (MNC velocity of the tibial nerve).

## 4. Discussion

In this study, we found correlations between electromyography and nerve conduction measurements among COVID-19 patients and the severity of the initial infection. Mainly, all three MNC measures of the median nerve correlated with the WHO clinical progression scale and two of the MNC measures of the median nerve correlated with the duration of acute hospitalization. Furthermore, we found strong correlations between the MNC amplitude of all tested nerves and the duration of rehabilitation. Furthermore, greater MNC amplitude was correlated with a higher FIM at discharge. Similar correlations were found between most SNC measures and the duration of rehabilitation and FIM at discharge. Finally, we found that the MNC latency of the median and the peroneal nerves and the MNC velocity of the median and tibial nerves predict 91.6% of the variance of the motor FIM at discharge.

The population that participated in this study show neuromuscular manifestations that are similar to those documented in the current literature. In our study, 47.4% of our subjects were diagnosed with myopathy. In a similar study that performed neurophysiological evaluations on COVID-19 patients (*n* = 21), abnormal findings were reported in 81% of their subjects [5]. Interestingly, myopathy was also found in 11 (55%) COVID-19 patients that were examined due to sensory symptoms [27] and 6 (50%) COVID-19 patients that were asymptomatic for muscular involvement [28]. This reoccurrence of myopathy in COVID-19 patients, as shown in our study, is well-documented in the literature since the COVID-19 outbreak.

Approximately 75% of our subjects, most of them ventilated during intensive care, had abnormal MNC amplitude of the peroneal nerve. It was suggested that compressive unilateral peroneal neuropathy might result from unconventional use of prone ventilation, which is meant to improve oxygenation and reduce ventilatory lung injury [29]. Upper limb nerves, e.g., the ulnar nerve, were also found to be affected by prolonged prone positioning during ventilation [6,30]. This might explain our results of the high prevalence of impaired nerve conduction of the lower limb nerve as well as the ulnar nerve that had reduced MNC amplitude in almost half of our subjects.

The tibial nerve was previously reported as the main location of the COVID-19 neuropathy in 10 COVID-19 patients [31]. Other affected nerves were the peroneal, median and ulnar nerves [31]. To the best of our knowledge, our study is the first to report that high MNC latency and low MNC amplitude and velocity of the median nerve are strongly associated with higher scores of the WHO clinical progression scale, indicating the worse severity of the illness. Higher MNC amplitude of the tibial nerve are also associated with this score. Clinicians should be aware of this association between the severity of the COVID-19 illness and the nerve conduction abnormalities, so that patients with higher WHO clinical progression scale scores can receive early examinations and adequate rehabilitation treatment for their condition.

The rehabilitation outcomes in this study present good efficacy of the provided rehabilitation program, as both cognitive and motor abilities of the lower and upper body were significantly improved. Predominantly, the gait velocity and endurance were improved in the majority of our subjects. Fatigue is an imperative symptom affecting patients with chronic respiratory diseases. In a recent mapping review regarding physical performance measures in COVID-19 patients [32], 6 studies that measured the 6MWT in the rehabilitation settings (total of 170 subjects) reported a range of mean values between 45 m and 323 m. The 2 large sample studies in the aforementioned review reported 6MWT means of 159 m (*n* = 72) and 229 m (*n* = 42). Our population improved their 6MWT scores from 180 m to 335 m, values that are comparable with the reported literature. So although our study did not include a control group, due to ethical reasons, we believe that the rehabilitation intervention provided to our subjects is similar to that provided to COVID-19 patients worldwide.

Since nerve conduction studies of COVID-19 patients were mostly case studies, the correlation between these measures and the rehabilitation outcomes have yet to be published. We report that a greater MNC amplitudes of all of the examined nerves were correlated with a shorter duration of rehabilitation and higher FIM at discharge. This finding is further strengthened by our regression analysis, showing that almost all of the variability of the FIM at discharge is explained by MNC latency of the median and the peroneal nerves and the MNC velocity of the median and tibial nerves. This new information contributes to our understanding regarding the large disparity within COVID-19 patients and the suggested importance of early electro-diagnostics in COVID-19 patients, especially those with severe illness. Previous studies, following the Severe Acute Respiratory Syndrome (SARS) outbreak, report that although physical function is increased within the first 6 months following infection onset, patients still experience residual impairments in physical function, even two years after the infection [33]. If awareness to the association between impairment of nerve conduction and rehabilitation outcomes is increased, then emphasis on an optimal treatment plan designed accordingly might produce better long-term prognosis for these patients. The application of focal vibration on tendons or muscles should be considered and integrated into clinical practice in order to develop suitable rehabilitation programs and improve neuromuscular functions, as previously evaluated for the treatment of several neuro-motor disorders [34].

The main study limitations include the small sample size. Moreover, the effects of drugs and other confounders, e.g., comorbidities, were not accounted for in the statistical analyses. It is possible that the illness worsened already existing symptoms that were not reported by the subjects during recruitment for this study. Additionally, most of our subjects were men, who are more affected by COVID-19 than woman [35]. Another possible limitation that might explain the lack of association between the WHO clinical progression scores and the SNC measurements is that we did not use standardized evaluation tools to distinguish between nociceptive and neuropathic pain. Finally, some of the reported MCID values might be underestimated since they were acquired for older populations or individuals post-stroke.

## 5. Conclusions

In summary, this is the first study showing correlation between nerve conduction abnormalities and severity of COVID-19 infection as well as rehabilitation outcomes. We conclude that electro-diagnostic studies, especially in COVID-19 patients with severe illness, are important in order to evaluate the prognosis and to develop suitable rehabilitation programs in order to improve neuromuscular functions, to restore daily functions and to improve independence of these patients. Future longitudinal studies should include follow-up examinations of the functional and electrophysiological status of these patients.

## Figures and Tables

**Table 1:** Number of subjects (percentage of total *n* = 19 in parentheses) and number of subjects with myopathy (percentage of *n* = 9 in parentheses) with pathological electrophysiological measurements of the Motor Nerve Conduction (MNC) and Sensory Nerve Conduction (SNC).

Measure	Wrist				Ankle					
	Median Nerve		Ulnar Nerve		Sural Nerve		Peroneal Nerve		Tibial Nerve	
	Total (*n* = 19)	With Myopathy (*n* = 9)	Total (*n* = 19)	With Myopathy (*n* = 9)	Total (*n* = 19)	With Myopathy (*n* = 9)	Total (*n* = 19)	With Myopathy (*n* = 9)	Total (*n* = 19)	With Myopathy (*n* = 9)
MNC latency (ms)	5 (26.3%)	2 (22.2%)	4 (21.1%)	1 (11.1%)	-	-	5 (26.3%)	2 (22.2%)	3 (15.8%)	1 (11.1%)
MNC velocity (m/s)	3 (15.8%)	1 (11.1%)	4 (21.1%)	2 (22.2%)	-	-	7 (36.8%)	6 (66.7%)	10 (52.6%)	6 (66.7%)
MNC amplitude (mV)	5 (26.3%)	2 (22.2%)	9 (47.4%)	4 (44.4%)	-	-	14 (73.7%)	7 (77.8%)	13 (68.4%)	8 (%88.9)
SNC velocity (ms)	5 (26.3%)	5 (55.6%)	7 (36.8%)	4 (44.4%)	10 (52.6%)	6 (66.7%)	-	-	-	-
SNC amplitude (µV)	5 (26.3%)	3 (33.3%)	6 (31.6%)	4 (44.4%)	11 (57.9%)	7 (77.8%)	-	-	-	-

**Table 2:** Median and interquartile range of the functional independence measure (FIM), grip force for each hand, the 6 minute walk test (6MWT) and 10 m walk test (10MWT) (*n* = 19). Data are presented at baseline and at discharge. The significance level, p, and effect size, r, are shown.

Measure	Baseline	At Discharge	p	r
Cognitive FIM	33 (30–35)	34 (31–35)	0.011	−0.580
Motor FIM	75 (66–88)	89 (84–90)	<0.001	−0.815
General FIM	109 (100–119)	121 (116–123)	<0.001	−0.855
Hand grip force (%)	41.5 (16.6–49.5)	51.5 (34.0–74.5)	<0.001	−0.854
6MWT (m)	180 (100–280)	335 (285–429)	<0.001	−0.877
10MWT (s)	13 (9–16)	8 (8–10)	<0.001	−0.857

**Table 3:** Spearman’s correlation coefficient and significance level (r, p) found between the WHO clinical progression scale and the duration of acute hospitalization (in weeks) and Motor Nerve Conduction (MNC) and Sensory Nerve Conduction (SNC) measures. Significant correlation are in bold font.

Parameter Type	Nerve	WHO Clinical Progression Scale	Weeks of Acute Hospitalization
MNC latency (ms)	Median nerve	**0.595, 0.009**	**0.517, 0.049**
	Ulnar nerve	0.233, 0.353	0.071, 0.802
	Peroneal nerve	−0.064, 0.800	−0.281, 0.310
	Tibial nerve	−0.042, 0.870	0.009, 0.975
MNC velocity (m/s)	Median nerve	**−0.563, 0.012**	−0.008, 0.976
	Ulnar nerve	−0.113, 0.644	−0.143, 0.598
	Peroneal nerve	−0.131, 0.593	−0.218, 0.416
	Tibial nerve	−0.049, 0.841	−0.092, 0.736
MNC amplitude (mV)	Median nerve	**−0.474, 0.040**	**−0.511, 0.043**
	Ulnar nerve	−0.138, 0.572	−0.220, 0.414
	Peroneal nerve	−0.233, 0.336	−0.429, 0.097
	Tibial nerve	**−0.489, 0.034**	−0.255, 0.340
SNC velocity (ms)	Median nerve	−0.193, 0.428	−0.313, 0.238
	Ulnar nerve	−0.101, 0.692	−0.179, 0.506
	Sural nerve	−0.101, 0.691	−0.179, 0.506
SNC amplitude (µV)	Median nerve	−0.428, 0.068	−0.077, 0.778
	Ulnar nerve	−0.139, 0.570	0.095, 0.726
	Sural nerve	−0.298, 0.216	−0.283, 0.288

**Table 4:** Significant Spearman’s correlation coefficient, r, found between the electrophysiological measurements, the Motor Nerve Conduction (MNC) and Sensory Nerve Conduction (SNC), and factors of the rehabilitation durations, as well as measurements at discharge of functional independence measure (FIM), 6 minute walk test (6MWT), and 10 m walk test (10MWT). Significant correlation are in bold font.

Parameter Type	Nerve	Months of Rehabilitation	Grip Force	Motor FIM	6MWT	10MWT
MNC latency (ms)	Median nerve	0.464, 0.053	0.210, 0.403	−0.246, 0.326	−0.160, 0.525	0.301, 0.225
	Ulnar nerve	0.227, 0.365	−0.015, 0.953	−0.244, 0.329	−0.195, 0.439	0.215, 0.391
	Peroneal nerve	0.014, 0.956	0.177, 0.483	−0.237, 0.344	−0.141, 0.578	−0.171, 0.497
	Tibial nerve	−0.111, 0.660	0.074, 0.771	**−0.493, 0.038**	−0.425, 0.079	0.290, 0.244
MNC velocity (m/s)	Median nerve	−0.376, 0.122	0.017, 0.946	**0.540, 0.017**	0.008, 0.974	0.028, 0.909
	Ulnar nerve	−0.063, 0.797	0.233, 0.337	0.299. 0.213	−0.121, 0.623	0.371, 0.118
	Peroneal nerve	−0.377, 0.111	−0.093, 0.705	0.100, 0.685	0.039, 0.875	**−0.462, 0.047**
	Tibial nerve	−0.199, 0.414	−0.014, 0.956	**0.530, 0.020**	0.278, 0.249	−0.321, 0.181
MNC amplitude (mV)	Median nerve	**−0.609, 0.006**	0.183, 0.453	**0.525, 0.021**	0.203, 0.405	0.025, 0.918
	Ulnar nerve	**−0.627, 0.004**	0.000, 0.999	0.377, 0.112	0.379, 0.110	−0.341, 0.153
	Peroneal nerve	**−0.534, 0.019**	0.181, 0.459	**0.590, 0.008**	0.423, 0.071	−0.056, 0.820
	Tibial nerve	**−0.479, 0.038**	0.075, 0.760	**0.535, 0.018**	0.161, 0.511	−0.370, 0.119
SNC velocity (ms)	Median nerve	**−0.594, 0.007**	0.161, 0.509	**0.599, 0.007**	0.384, 0.104	**−0.476, 0.040**
	Ulnar nerve	**−0.475, 0.046**	0.057, 0.822	**0.523, 0.026**	0.371, 0.129	−0.229, 0.360
	Sural nerve	−0.402, 0.088	0.193, 0.429	0.397, 0.092	0.156, 0.524	−0.301, 0.210
SNC amplitude (µV)	Median nerve	**−0.562, 0.012**	−0.089, 0.718	0.285, 0.237	0.064, 0.796	0.082, 0.738
	Ulnar nerve	−0.017, 0.945	0.250, 0.302	0.089, 0.718	−0.212, 0.383	0.189, 0.439
	Sural nerve	−0.391, 0.098	0.141, 0.566	**0.463, 0.046**	0.244, 0.315	−0.127, 0.605

## Data Availability

Data are contained within the article or Supplementary Material.

## Supplementary Materials

The following supporting information can be downloaded at: https://www.mdpi.com/article/10.3390/biology11020277/s1, SPSS file.
